# Drug resistance in bacteria isolated from patients presenting with wounds at a non-profit Surgical Center in Phnom Penh, Cambodia from 2011–2013

**DOI:** 10.1186/s40794-015-0006-5

**Published:** 2015-07-31

**Authors:** Boren Hout, Chamroeun Oum, Putheavy Men, Vanvathanak Vanny, Chonthida Supaprom, Vireak Heang, Agus Rachmat, Michael Prouty, Steven Newell, Dustin Harrison, Saqib Noor, James Gollogly, Ly Tho, Yong June Kim, Gavin Ford

**Affiliations:** 1 Naval Medical Research Unit - No.2, Phnom Penh, Cambodia; 2 Children’s Surgical Center, Phnom Penh, Cambodia; 3 Department of Infectious Diseases, Walter Reed National Military Medical Center, Bethesda, MD USA

**Keywords:** Bacteria, Wound infections, Microbial drug resistance, Cambodia

## Abstract

**Background:**

Emerging antibiotic resistance amongst clinically significant bacteria is a public health issue of increasing significance worldwide, but it is relatively uncharacterized in Cambodia. In this study we performed standard bacterial cultures on samples from wounds at a Non-Governmental-Organization (NGO) Hospital in Phnom Penh, Cambodia. Testing was performed to elucidate pathogenic bacteria causing wound infections and the antibiotic resistance profiles of bacterial isolates. All testing was performed at the Naval Medical Research Unit, No.2 (NAMRU-2) main laboratory in Phnom Penh, Cambodia.

**Methods:**

Between 2011–2013, a total of 251 specimens were collected from patients at the NGO hospital and analyzed for bacterial infection by standard bacterial cultures techniques. Specimens were all from wounds and anonymous. No specific clinical information accompanied the submitted specimens. Antibiotic susceptibility testing, and phenotypic testing for extended-spectrum beta-lactamase (ESBL) were performed and reported based on CLSI guidelines. Further genetic testing for CTX-M, TEM and SHV ESBLs was accomplished using PCR.

**Results:**

One-hundred and seventy-six specimens were positive following bacterial culture (70 %). *Staphlycoccus aureus* was the most frequently isolated bacteria. Antibiotic drug resistance testing revealed that 52.5 % of *Staphlycoccus aureus* isolates were oxacillin resistant. For *Escherichia coli* isolates, 63.9 % were ciprofloxacin and levofloxacin resistant and 96 % were ESBL producers. Resistance to meropenem and imipenem was observed in one of three *Acinetobacter spp* isolates.

**Conclusions:**

This study is the first of its kind detailing the antibiotic resistance profiles of pathogenic bacteria causing wound infections at a single surgical hospital in Cambodia. The reported findings of this study demonstrate significant antibiotic resistance in bacteria from injured patients and should serve to guide treatment modalities in Cambodia.

## Background

Providing effective health care, in developing countries such as Cambodia is challenged by the spread of drug resistant pathogens [1]. Emergent resistant pathogenic strains have demonstrated potential to quickly spread beyond their initial geographic point of origin [2]. Well documented examples include the spread of specific methicillin-resistant *S. aureus* (MRSA) clones around the world capable of outcompeting pre-existing local populations [3, 4]; the emergence of *Enterobacteriaceae* with resistance to carbapenems conferred by New Delhi metallo-β-lactamase 1 (NDM-1) from India and Pakistan [5]; and the global spread of multidrug resistant *Acinetobacter* species [6]. Another factor that fuels antibiotic resistance in Cambodia is the unregulated access to antibiotics. In addition, the quality of antibiotics is not well regulated with a significant presence of counterfeit drugs on the market [7, 8].

Data on antibiotic drug resistance amongst clinical significant bacteria in Cambodia is limited to date. The significance of the development of drug resistant motifs was evidenced in an investigation of the prevalence of CTX-M beta-lactamase enzymes in *E. coli* causing community acquired urinary tract infections [9]. Another study reported on the first documented cases of community acquired Methicillin Resistant *S. aureus* (MRSA) infections in Cambodia. In this work, seventeen children were found to have community acquired MRSA infections. All cases were caused by two independent MRSA clones identified by molecular characterization [10]. A recent large study into bacterial causes of blood stream infections at a community hospital in Phnom Penh from 2007–2010 demonstrated high level antibiotic resistance patterns to include an observed 62.3 % resistance of *E. coli* isolates to ciprofloxacin, 90 % of *Salmonella typhi* isolates having decreased susceptibility to ciprofloxacin, and 21.7 % of *S. aureus* isolates being resistance to methicillin [11].

The health care infrastructure in Cambodia is often not accessible to people who lack the means to pay resulting in inadequate care [12]. This situation can lead to the development of chronic wounds associated with traumatic injuries that remain untreated and which often develop polymicrobic infections resulting in poor outcomes.

In this study we focus on the etiologic causes of wound infections at a Non-Governmental-Organization (NGO) surgical hospital in Phnom Penh, Cambodia, and describe the drug resistance profiles of those agents. Our findings demonstrate significant antibiotic resistance in bacteria from injured patients.

## Methods

### Sample collection

The Children’s Surgical Center (CSC) is a non-governmental organization (NGO) located in Phnom Penh, Cambodia that triages between 10,000-14,000 patients per year in all age groups. The center serves as a terminal medical facility receiving patients from across Cambodia. Wounds seen include both traumatic wounds (vehicle accident, deep tissue burns, general lacerations) as well as surgical wounds resulting from infections from prior surgeries.

### Culture

Samples were collected as part of routine clinical practice by CSC personnel. Samples of wound exudates were taken from the most seriously ill patients and placed in standard culture tubes containing bacterial transport media. One swab culture was taken from each wound and placed in Stuarts bacterial transport media. Samples were sent with a corresponding specimen transport form.

Samples were inoculated on blood agar, Colistin Nalidixic Acid Agar, and MacConkey agar for 48 hours. Direct gram staining was performed on all samples that came from closed wounds. For positive cultures, gram staining and identification using standard biochemical analyses were carried out to identify the species of isolated bacteria (API®, Biomérieux; http://www.biomerieux-usa.com/servlet/srt/bio/usa/dynPage?open=USA_PRD_LST&doc=USA_PRD_LST_G_PRD_USA_5&pubparams.sform=0&lang=en). Antibiotic susceptibility testing (AST) was done using the Kirby-Bauer method [13] and Epsilometer test (E-test) method on pathogenic organisms. ASTs were interpreted in accordance with the antibiotic susceptibility break-points published by the Clinical Laboratory Standards Institute (CLSI) [14, 15]. Results were reported back to CSC by electronic mail. AST results were reported in terms of Sensitive (S), Intermediate (I), and Resistant (R) based on existing CLSI guidelines.

### ESBL testing

Phenotypic screening was performed on gram negative bacteria suspected of expressing Extended-Spectrum Beta-lactamases (ESBLs). *E. coli, Klebsiella spp.* and *Proteus mirabilis* isolates showing reduced susceptibility to ceftriaxone (CRO 30 μg, zone diameter of ≤ 25 mm), ceftazidime (CAZ 30 μg, zone diameter of ≤ 22 mm), aztreonam (ATM 30 μg, zone diameter of ≤ 27 mm) were selected for ESBL confirmatory testing as per CLSI guidelines (*20*). Phenotypic confirmation disk diffusion testing (PCDD) was performed on Mueller Hinton agar. Ceftazidime (CAZ 30 μg), ceftazidime with clavulanic acid (CAZ/CLA 30/10 μg), cefotaxime (CTX 30 μg), and cefotaxime with clavulanic acid (CTX/CLA 30/10 μg) were used. These antibiotics were obtained from Becton Dickinson, USA. A greater than 5 mm increase in the zone of inhibition for the CAZ/CLA and CTX/CLA containing disk versus the corresponding CAZ or CTX disk was considered positive for ESBL [16].

### PCR

**Fig. 1:**
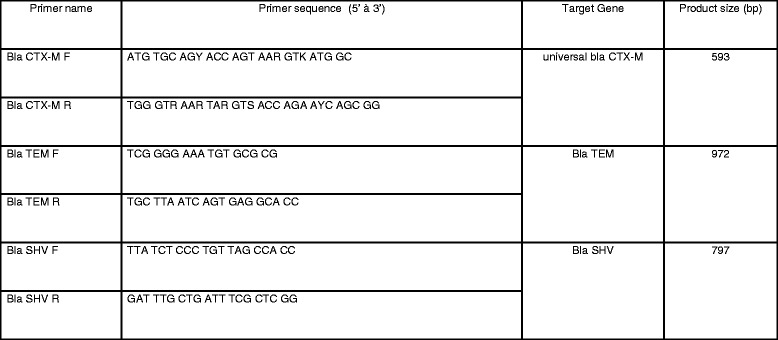
Primers used for ESBL PCR testing; Primer names followed by the primer base pair sequences reported in 5’ to 3’ direction for each target ESBL gene, and the resulting product size in base pairs (bp)

PCR based genetic detection of CTX-M, TEM and SHV ESBLs was conducted on extracted DNA from bacterial isolates. DNA was extracted from the isolates by QIAamp DNA Mini Kit (cat. # 51306). A total of 2 ul of extracted DNA was used as a template for PCR analysis. GenAmp PCR core reagent (ABI, cat. # N808-0009) was used to amplify the sample DNA by mix with a specific primer set (Fig. 1) of CTX-M, TEM and SHV. PCR was performed using primers and thermocycling conditions as in ref [17, 18].

### Data management and analysis

Samples were identified by unique numerical identifiers. Data was double-entered and automatically reviewed with a program that detects errors in consistency. Data was collected, stored, and managed through the execution of the study using MS Access® (Microsoft Inc., Redmond, WA, USA).

## Results

Standard aerobic cultures of 251 wound specimens revealed 176 positive samples (70 %), with 93 (53 %) having a single pathogen and 83 (47 %) having multiple pathogens. *S. aureus* was the most frequently isolated bacteria with 99 isolates (56 %). The following bacteria are listed in terms of decreasing frequency of isolation: Coagulase-negative *Staphylococcus spp.* (CNSS) (21 %; 38 isolates); *E. coli* (20 %; 36 isolates); *Pseudomonas aeruginosa* (14 %; 24 isolates); *Enterobacter cloacae* (7 %; 13 isolates); *Proteus mirabilis* (7 %; 12 isolates); *Streptococcus pyogenes* (7 %; 12 isolates); *Klebsiella pneumoniae* (6 %; 10 isolates). All isolated bacteria are detailed in Table 1. A select antibiogram for isolated bacteria of interest is provided in Table 2. These bacteria were selected for their high clinical relevance. There was no resistance to the carbapenems meropenem or imipenem with the exception of a single *A. baumanni/calcoaceticus* isolate.

**Table 1:** Isolated bacteria from biological samples

No.	Isolated bacteria	No. of isolate
1	*Staphylococcus aureus*	99
2	*Coagulase Negative Staphylococcus sp*	38
3	*Escherichia coli*	36
4	*Pseudomonas aeruginosa*	24
5	*Enterobacter cloacae*	13
6	*Proteus mirabilis*	12
7	*Streptococcus pyogenes*	12
8	*Klebsiella pneumoniae*	10
9	*Raoultella terrigena*	8
10	*Morganella morganii*	7
11	*Burkholderia cepacia*	6
12	*Aeromonas hydrophila/caviae*	5
13	*Streptococcus constellatus*	5
14	*Enterococcus faecalis*	3
15	*Streptococcus agalactiae*	3
16	*Acinetobacter baumannii/calcoaceticus*	3
17	*Bacillus sp*	2
18	*Corynebacterium group G*	2
19	*Enterococcus avium*	2
20	*Klebsiella oxytoca*	2
21	*Providencia rettgeri*	2
22	*Streptococcus anginosus*	2
23	*Streptococcus equinus*	2
24	*Streptococcus oralis*	2
25	*Aeromonas hydrophila/ caviae*	1
26	*Alcaligenes faecalis*	1
27	*Burkholderia pseudomallei*	1
28	*Citrobacter koseri/farmeri*	1
29	*Citrobacter youngae*	1
30	*Corynebacterium strium/ amycolatum*	1
31	*Edwardsiella tarda*	1
32	*Enterobacter aerogenes*	1
33	*Enterobacter avium*	1
34	*Group D Beta-Hemolytic Streptococci*	1
35	*Group G Beta-Hemolytic Streptococci*	1
36	*Leuconostoc spp*	1
37	*Pantoea spp*	1
38	*Proteus vulgaris*	1
39	*Pseudomonas fluorescens*	1
40	*Pseudomonas luteola*	1
41	*Pseudomonas stutzeri*	1
42	*Ralstonia pickettii*	1
43	*Raoultella ornithinolytica*	1
44	*Rhodococcus spp*	1
45	*Serratia liquefaciens*	1
46	*Streptococcus dysgalactiae ssp equisimilis*	1
47	*Streptococcus mitis*	1

**Table 2:** Antibiogram for isolated bacteria of interest

Susceptility testing	*A. baumannii/ calcoaceticus*	Coag Neg *Staph spp*	*E. cloacae*	*E. coli*	*K. pneumoniae*	*P. mirabilis*	*P. aeruginosa*	*S. aureus*	*S. pyogenes*
	3	38	13	36	10	12	24	99	12
Amikacin: AK	S(2), R(1)		S(13)	R(1), I(1), S(34)	I(1), S(9)	S(12)	S(24)		
Amoxicillin-Clavulanic Acid: AmC			R(13)	R(6), I(7), S(23)	R(3), I(1), S(6)	R(4), S(7), I(1)			
Ampicillin: AM			R(12), I(1)	R(33), I(1), S(2)	R(10)	R(10), S(2)			
Ampicillin-Sulbactam: SAM	S(2), I(1)		R(8), S(5)	R(2), I(14), S(20)	R(4), S(6)	R(3), I(2), S(7)			
Aztreonam: ATM			R(1), S(10), I(2)	R(17), I(7), S(12)	R(5), S(5)	S(12)	R(3), I(4), S(17)		
Cefazolin: CZ			R(13)	R(25), S(11)	R(5), S(5)	R(3), I(4), S(5)			
Cefepime: FEP	R(2), S(1)		R(1), S(12)	R(8), I(10), S(18)	R(2), I(3), S(5)	S(10), I(2)	R(2), S(22)		R(1), S(11)
Cefoxitin: FOX		R(5), S(2)	R(13)	R(1), I(3), S(32)	R(1), S(9)	S(12)		R(52), S(47)	
Ceftazidime: CAZ	R(2), S(1)		S(12), I(1)	R(5), I(7), S(24)	R(2), I(2), S(6)	R(1), S(11)	R(3), S(21)		
Ceftriaxone: CRO	R(2), I(1)		R(3), S(9), I(1)	R(24), I(1), S(11)	R(5), S(5)	R(1), I(1), S(10)	R(9), I(9), S(6)		R(2), S(10)
Cefuroxime (Sodium): CXM			R(4), S(9)	R(25), S(11)	R(5), S(5)	R(3), S(9)			
Chloramphenicol: C		R(3), S(4)	R(4), S(9)	R(20), S(16)	R(6), S(4)	R(9), S(3)		R(7), S(92)	R(5), I(4), S(3)
Ciprofloxacin: CIP	R(2), S(1)	R(3), S(3), I(1)	R(2), I(2), S(9)	R(25), S(11)	R(2), I(3), S(5)	R(6), I(1), S(5)	R(4), S(20)	R(55), S(44)	
Clindamycin: CC		R(4), S(1), I(2)						R(53), I(7), S(39)	R(1), I(3), S(8)
Erythromycin: E		R(6), S(1)						R(68), I(8), S(23)	R(5), I(2), S(5)
Gentamicin: GM	R(2), S(1)	R(3), S(2), I(2)	R(2), S(11)	R(22), I(2), S(12)	R(4), S(6)	R(8), S(4)	R(5), S(19)	R(48), S(51)	
Imipenem: IPM	R(1), S(2)		S(13)	S(36)	S(10)	S(12)	S(24)		
Levofloxacin: LVX	R(1), S(1), I(1)	R(3), S(3), I(1)	R(2), S(11)	R(23), I(2), S(11)	R(2), S(8)	R(4), I(2), S(6)	R(5), S(19)	R(53), I(2), S(44)	I(2), S(10)
Meropenem: MEM	R(1), S(2)		S(13)	S(36)	S(10)	S(12)	S(24)		
Oxacillin : OX		R(5), S(2)						R(52), I(3), S(44)	
Penicillin: P		R(6), S(1)						R(98), S(1)	R(1), S(11)
Piperacilin: PIP	R(2), S(1)		R(4), S(9)	R(32), I(2), S(2)	R(6), I(2), S(2)	R(4), I(2), S(6)	R(4), S(20)		
Tetracycline: Te	R(2), S(1)	R(5), S(2)	R(6), S(7)	R(32), S(4)	R(7), S(3)	R(11), S(1)		R(36), I(1), S(62)	R(10), I(1), S(1)
Tobramycin: NN, TM	R(1), (S(2)						R(4), I(1), S(19)		
Trimethoprim-Sulfamethoxazole: SXT	R(2), S(1)	R(4), S(3)	R(5), S(8)	R(28), I(1), S(7)	R(5), S(5)	R(8), I(1), S(3)		R(22), I(5), S(72)	
Vancomycin: Va		S(7)						S(99)	S(12)

*I* Intermediate, *R* Resistant, *S* Sensitive

**Fig. 2:**
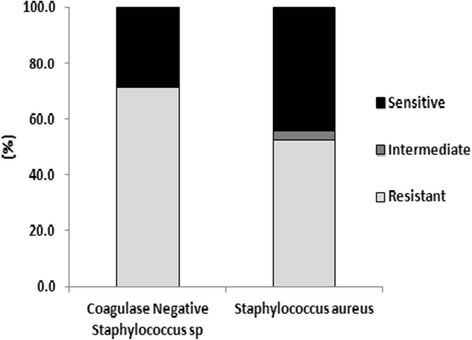
*S. aureus* and CNSS resistance to oxacillin; Relative percentages of Coagulase-negative *Staphylococcus spp.* (CNSS) and *S. aureus* isolates that were sensitive, intermediate, and resistant to oxacillin

Fifty-two of 99 (52.5 %) *S. aureus* isolates were oxacillin resistant (MRSA). Of the 52 MRSA isolates, some maintained susceptibility to trimethoprim-sulfamethoxazole (32 isolates); tetracycline (27 isolates); and clindamycin (8 isolates). Only one MRSA isolate was sensitive to all three of these antibiotics, while 3 MRSA isolates were resistant to all three. Of the 38 CNSS isolates identified, 7 isolates underwent AST before a change in procedures classified such species of *Staphylococcus* as non-pathogenic for the remainder of the study. Of the 7 CNSS isolates that underwent AST, 5 isolates demonstrated resistance to oxacillin. Rates of *S. aureus* and CNSS resistance to oxacillin is represented in Fig. 2.

Other isolated gram positive bacteria of clinical significance included *S. pyogenes* and *E. faecalis*. There were 12 *S. pyogenes* isolates, 11 of which were sensitive to penicillin, and one that was resistant (confirmed but unable to perform additional testing due to resource constraints). There were 3 *E. faecalis*, all of which were sensitive to ampicillin and vancomycin.

**Fig. 3:**
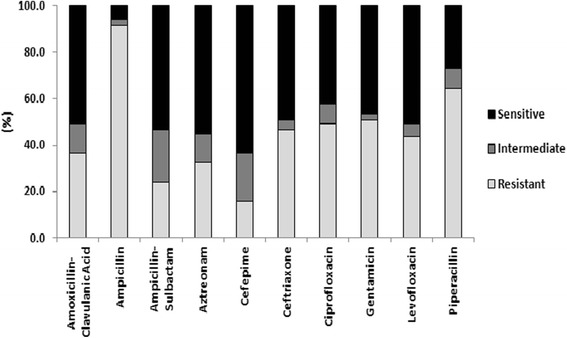
*E. coli*/*E. cloacae*/*P. mirabilis*/*K. pneumoniae* group antibiotic resistance; The relative percentages of these four *Enterobacteriaceae* that were sensitive, intermediate, and resistant to commonly used antibiotics with variable degrees of activity against gram negative bacteria

*E. coli, Enterobacter cloacae, Proteus mirabilis,* and *Klebsiella pneumoniae* demonstrated significant antibiotic resistance as shown in Table 2 and Fig. 3. These are all *Enterobacteriaceae* that are common causative pathogens to clinical bacterial disease. They were also all isolated at a relatively high frequency in this study making an analysis of their associated antibiotic trends more significant. There were a total of 71 isolates for those 4 species. Thirty of 71(42.3 %) isolates were resistant to both ciprofloxacin and levofloxacin. Twenty-three of 36 *E. coli* (63.9 %) isolates had resistance to both levofloxacin and ciprofloxacin. Four of 24 (16.6 %) *P. aeruginosa* isolates demonstrated resistance to levofloxacin and ciprofloxacin.

For the group of four species of gram negative bacteria, 65 of 71 (91.5 %) isolates were resistant to ampicillin. Of these 65 isolates, only 24 isolates maintained sensitivity to amoxicillin-clavulanic acid and ampicillin-sulbactam. A total of 45 of 71 (63.4 %) isolates were resistant to piperacillin. Four of 24 (16.6 %) *P. aeruginosa* isolates were resistant to piperacillin. The beta-lactamase inhibitor combination antibiotic piperacillin-tazobactam was not tested in this study because it was not available. *P. aeruginosa* isolates were not tested against ampicillin, amoxicillin-clavulanic acid, or ampicillin-sulbactam as per CLSI guidelines [14, 15].

Amongst the highlighted aerobic gram negative bacteria, 36 of 71 (50.7 %) isolates were resistant to the aminoglycoside gentamicin. Only one *E. coli* isolate was resistant to amikacin, while a *K. pneumonia*e isolate had intermediate resistance to amikacin. A total of 23 of 71 (32.4 %) isolates were resistant to the monobactam aztreonam. Three of 24 (12.5 %) isolates for *P. aeruginosa* were resistant to aztreonam, and 5 of 24 (20.8 %) isolates were resistant to gentamicin, respectively. There was no observed *P. aeruginosa* resistance to amikacin.

A total of 33 of 71 (46.5 %) gram negative bacterial isolates of interest were resistant to ceftriaxone. *P. aeruginosa* isolates had a 12.5 % and 8.3 % rate of resistance to the anti-pseudomonal cephalosporins ceftazidime and cefepime, respectively. The group resistance of gram negative bacterial isolates of interest to selected antibiotics is represented in Fig. 3.

**Fig. 4:**
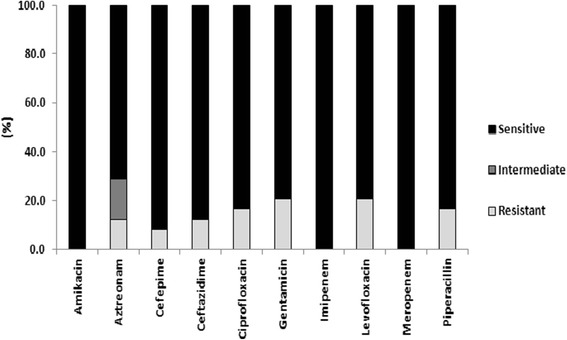
*P. aeruginosa* antibiotic resistance; The relative percentages of *P. aeruginosa* isolates that were sensitive, intermediate, and resistant to common antibiotics with variable degrees of anti-pseudomonas activity

Sensitivity testing was performed on *P. aeruginosa* against the known anti-pseudomonal antibiotics pipericillin, aztreonam, ceftazidime, cefepime, gentamicin, amikacin, ciprofloxacin, levofloxacin, meropenem, and imipenem. The rates of resistance for *P. aeruginosa* and those antibiotics were as follows: pipericillin (16.6 %); aztreonam (12.5 % resistant ); ceftazidime (12.5 %); cefepime (8.3 %); gentamicin (20.8 %); amikacin (0 %); ciprofloxacin (16.6 %); levofloxacin (20.8 %); meropenem (0 %); imipenem (0 %). The resistance of *P. aeruginosa* to particular anti-pseudomonal antibiotics is represented in Fig. 4.

**Fig. 5:**
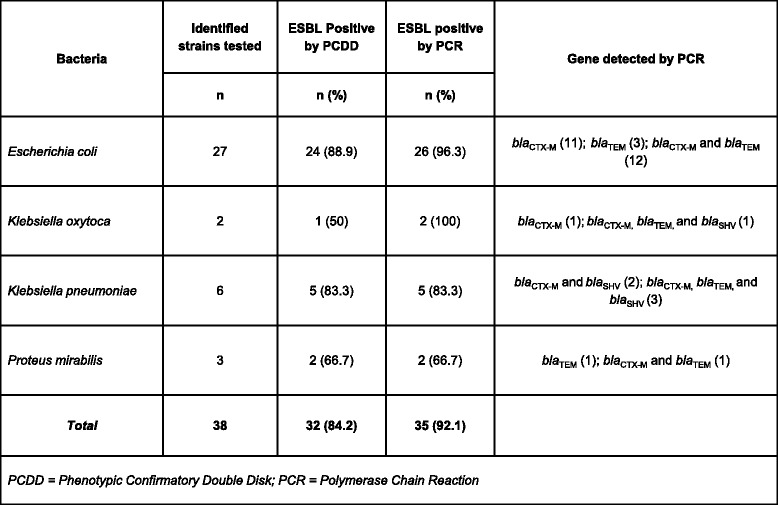
ESBL testing – PCDD and PCR results; *E. coli*, *K. oxytoca*, *K. pneumoniae*, and *P. mirabilis* isolates tested for presence of ESBLs by PCDD and PCR. N is the number of isolates tested by PCDD and PCR with the percentage positive in parentheses. The ESBL genes detected by PCR are listed for each species of bacteria tested with the number detected for each gene listed in parentheses

Twenty-seven of 36 *E. coli*, 2 of 2 *K. oxytoca*, 6 of 10 *K. pneumoniae*, and 3 of 12 *P. mirabilis* isolates, respectively, demonstrated phenotypic resistance to ceftriaxone, ceftazidime, or aztreonam by disk diffusion that can serve as an initial screening test for presence of ESBLs per CLSI guidelines [16]. PCDD using cefotaxime and ceftazidime alone and in combination with clavulanic acid was confirmatory for 32 of 38 (84.2 %) isolates. Results for PCR testing for CTX-M, TEM, and SHV ESBL genes in these 38 isolates is represented in (Fig. 5).

Three isolates, 2 *E.coli* and 1 *K. oxytoca*, were ESBL screen positive, but subsequently tested negative by PCDD. Genetic screening of these isolates revealed the presence of TEM genetic sequences in the 2 *E. coli*, and CTX-M genetic elements in the *K. oxytoca* isolate.

## Discussion

Successful treatment of wound infections requires laboratory analysis to identify the pathogen(s) causing the infection. However, in resource scarce countries such as Cambodia the ability to conduct routine diagnostic microbiological procedures is lacking. Throughout Cambodia antimicrobial susceptibility information is usually unavailable, and inappropriate antimicrobial therapy is often prescribed as a result. A study of three pilot health centers in Kampong Thom province reported multiple instances of inappropriate antimicrobial therapy, most significantly high antibiotic use (66-100 %), polypharmacy (2.35 per consultation), and the unnecessary use of injections (0.9-4.5 %) [19]. The appropriate use of antimicrobial therapy can decrease disease duration, prevent progression to severe disease, and increase successful resolution of infections, whereas overtreatment may exert selective pressure, potentially contributing to increasing antimicrobial-resistance levels [20].

In an effort to create a longitudinal data set on the bacterial causes of wound infections, we studied the aerobic bacterial causes of wound infections amongst patients at a single NGO surgical center in Phnom Penh. Such a data set is essential in order to better characterize emerging patterns of antibiotic resistance. Our study contributes to a better understanding of wound infections in resource limited countries like Cambodia, and can facilitate more informed and effective clinical practice and public health policy.

Our study demonstrated oxacillin resistance among 52 of 99 (52.5 %) *S. aureus* isolates. There have been multiple risk factors associated with the emergence of Methicillin Resistant *S. aureus* (MRSA) in remote settings such as: lack of access to clean water for frequent bathing, high frequency of anti-microbial drug consumption, and sub-standard living conditions with overcrowding [21, 22]. CSC is a highly skilled non-profit surgical center in the capital of Cambodia, with an indigent patient population. These risk factors for emergence of MRSA are relevant to this patient population. The US CDC has strict definitions for a community-acquired MRSA infection and for a hospital acquired infection (HAI) [23]. We cannot speculate as to whether these MRSA infections were contracted via community-acquired mechanisms or were the result of previous stays in other medical treatment facilities.

Specific clinical information relating to presentation, course, or outcome for patients anonymously involved in this study was not available. We believe the MRSA isolates recovered for this study represent a mix of community and hospital acquired infections in this highly indigent patient population. Frequently patients come to CSC when prior care at other clinics or hospitals has failed. This salvage therapy scenario is one means by which patients with MRSA infections, acquired through nosocomial transmission, may present for care at CSC. The designation of MRSA as community or hospital acquired is important for the purposes of surveillance of multi-drug resistant organisms (MDROs) as it characterizes where such pathogens are present in the geographic area of study. The characterization of MDROs and the degree to which they are causing community versus hospital acquired infections in Cambodia is an area deserving additional study both for the purposes of MDRO surveillance and for public health benefits.

Clindamycin, trimethoprim-sulfamethoxazole, and the tetracyclines are commonly used for coverage of skin and soft tissue infections caused by community-acquired MRSA. In oral formulations, they are a convenient way to manage such infections in the ambulatory setting. These antibiotics remain a recommendation for this indication by the Infectious Disease Society of America clinical practice guidelines [24]. Our results show reduced sensitivities of MRSA isolates to these antibiotics. Only thirty-two out of 52 isolates were sensitive to trimethoprim-sulfamethoxazole (61.5 %); 27 of 52 isolates sensitive to tetracycline (51.9 %); and 8 of 52 isolates sensitive to clindamycin (15.4 %). Such a situation represents a very challenging therapeutic dilemma for doctors and patients at a no-fee-for-service center such as CSC where parental antibiotics active against MRSA are prohibitively expensive.

Vancomycin resistance of *S. aureus* is a well known and concerning trend. Increased prevalence of MRSA and the subsequent increased use of vancomycin in the treatment of MRSA infections are two driving forces behind that trend [25]. There was no vancomycin resistance among *S. aureus* isolates in our study. The lack of access and relatively infrequent use of vancomycin could be a factor in the absence of decreased sensitivity of *S. aureus* isolates to that antibiotic.

The emergence of increasingly resistant gram negative bacteria presents a dire situation for infected patients and their treating physicians. The 6 ESKAPE pathogens (*E. faecium, S. aureus, K. pneunomiae, Acinetobacter* species, *P. aeruginosa,* and *Enterobacter* species) cause the majority of infections in US hospitals and are recognized as some of the most significant emerging infectious disease threats of this century. These bacteria frequently “escape” the effects of antibiotics. Four of the bacteria in this group are gram negative bacilli. Gram negative bacteria are important not only due to the numbers of infections caused, but also because substantial, even pan resistance to antibiotics is increasingly observed [26].

Currently, the number of unique beta-lactamases described from clinical isolates is estimated to be at least 1,300 [27]. Some mechanisms of resistance are an evolutionary response to the pressure of antibiotic use, while others have been demonstrated to have been present for thousands of years [28]. Clinicians are faced with examples of extreme resistance such as carbapenemase producing *Enterobacteriaceae* [5] and *K. pneumoniae* [29]. In some cases, the polymyxins such as colistin, an older class of antibiotics, can be employed as salvage therapy, oftentimes as combination therapy with newer antibiotics. This clinical scenario has generated a reluctant, but renewed interest in these antibiotics, which possess a much more unfavorable side effect profile than newer drugs [30]. In this study, only 1/3 *A. baumannii/calcoaceticus* demonstrated resistance to meropenem and imipenem.

The fluoroquinolones are an important class of antibiotics prized for their broad spectrum of activity and ease of use with equivalent bioavailability in oral versus parental forms. Emerging resistance to these antibiotics is limiting their usefulness, especially in Southeast Asian countries. *E. coli* resistance to ciprofloxacin increased from 45.1 % to 51 % from 2000–2005 in one study in Thailand [31], and *E. coli* isolates in our study had an even higher rate of resistance (69.4 %) to ciprofloxacin. The 71 total isolates of *E. coli*, *E. cloacae*, *P. mirabilis*, and *K. pneumoniae* had, as a group, a 42.3 % rate of resistance to both ciprofloxacin and levofloxacin. This alarming rate of resistance will further burden the healthcare infrastructure in Cambodia in the near term. The ease of horizontal gene transfer among gram negative bacteria makes the spread of resistance determinants a near certainty and continued surveillance is required to address this situation in order to develop strategies to mitigate the damage that will be caused.

The proliferation of Extended Spectrum Beta Lactamases (ESBLs) is a major factor in the antibiotic resistance situation. There is no consensus definition for an ESBL [32]. One common definition states that an ESBL is a beta-lactamase conferring resistance or reduced susceptibility to the oxymino-beta-lactams (ceftiaxone, cefotaxime, ceftazidime) and aztreonam (a monobactam), but is inhibited by β-lactamase inhibitors such as clavulanic acid [33]. Globally the most prominent ESBLs include the CTX-M-14 and CTX-M-15 enzymes, as well as TEM-1, TEM-2, and SHV-1 enzymes. Out of the 35 isolates that tested positive for the ESBL genes of interest by PCR, 16 isolates were positive for 1 gene of interest. Fifteen isolates possessed 2 genes, and 4 isolates had all 3 genes. These 4 isolates were all *Klebsiella* species. It is well known that bacteria can harbor multiple genes of resistance simultaneously. ESBL genes are located on large plasmids that can also house resistance genes for fluoroquinolones, aminoglycosides, and trimethoprim-sulfamethoxazole [34]. For the three isolates that were ESBL PCR positive, but subsequently tested negative by PCDD, there could be multiple genetic reasons for those findings. An unidentified resistance mechanism could have allowed the isolates to screen positive while mutations in essential promoter elements for transcription of the ESBL genes could result, ultimately, in the genes not being translated. Therefore, testing as negative in the PCCD test while containing ESBL genetic elements. Such as scenario could cause PCR (genotypic) positive testing but negative PCCD (phenotypic) testing. This could be one of several possible reasons for the findings.

Antibiotic coverage of *P. aeruginosa* is an important consideration in multiple clinical scenarios, particularly in the treatment of nosocomial infections. The number of antibiotics with instrinsic activity against *P. aeruginosa* is limited. While there were only 24 *P. aeruginosa* isolates the rates of resistance to some antibiotics are interesting. For example, the rate of resistance to ciprofloxacin was only 16.6 % among *P. aeruginosa* compared to 69.4 % among *E. coli* isolates. The relatively low rates of resistance among *P. aeruginosa* isolates to the anti-pseudomonal antibiotics tested may be a reflection of this indigent patient population’s lack of access to potent antibiotics. This sort of exposure is known to selectively pressure bacteria into resistant phenotypes. It would be interesting to compare the results of this study to a patient population of greater means and more access to care. Such a patient population may have higher rates of resistance.

Limitations of this study included the fact that only standard aerobic culturing was performed, samples came from a single center, samples were taken from the “most ill”, and we did not use any selection criteria, which cause selection bias towards greater rates of resistance. The hospital was admittedly a source for referral of nosocomial cases, which would also bias to a greater likelihood of resistance. Culturing for anaerobic bacteria was not performed, nor was more sophisticated molecular biological techniques capable of identifying bacterial strains not recoverable by standard culture methods. These methods were beyond the resource constraints of the current study. This study focused predominantly on phenotypic demonstrations of antibiotic resistance (AST). Finally, the lack of associated clinical data did not allow correlations of resistance patterns with outcomes or risk factors. Considering the limitations imposed, we were able to recover and identify a pathogen(s) in 176 out of 251 specimens, a positive result rate of 70.1 %.

## Conclusions

Our work demonstrates concerning rates of resistance among clinically significant bacterial pathogens, with >50 % MRSA amongst *S. aureus* isolates and 96 % of *E. coli* isolates being ESBL producers. These results highlight the urgent need to expand the diagnostic microbiology infrastructure in Cambodia to enable the characterization and tracking of resistance trends in real time. This will in turn enable improvements in the clinical care of bacterial infections, and the reduction of some of the pressures driving the spread of bacterial resistance.

## References

[1] van der Bij AK, Pitout JD. The role of international travel in the worldwide spread of multiresistant Enterobacteriaceae. J Antimicrob Chemother. 2012;67:2090–100. 10.1093/jac/dks214 22678728

[2] Dong J, Olano JP, McBride JW, Walker DH (2008). Emerging pathogens: challenges and successes of molecular diagnostics. J Mol Diagn..

[3] Grundmann H, Aanensen DM, van den Wijngaard CC, Spratt BG, Harmsen D, Friedrich AW (2010). Geographic distribution of *Staphylococcus aureus* causing invasive infections in Europe: a molecular-epidemiological analysis. PLoS Med..

[4] David MZ, Daum RS (2010). Community-associated methicillin-resistant *Staphylococcus aureus*: epidemiology and clinical consequences of an emerging epidemic. Clin Microbiol Rev..

[5] Lascols C, Hackel M, Marshall SH, Hujer AM, Bouchillon S, Badal R (2011). Increasing prevalence and dissemination of NDM-1 metallo-β-lactamase in India: data from the SMART study (2009). J Antimicrob Chemother..

[6] Peleg AM, Seifert H, Paterson DL (2008). *Acinetobacter baumannii*: emergence of a successful pathogen. Clin Microbiol Rev..

[7] Lon CT, Tsuyuoka R, Phanouvong S, Nivanna N, Socheat D, Sokhan C (2006). Counterfiet and substandard antimalarial drugs in Cambodia. Trans R Soc Trop Med Hyg..

[8] Sokheng V. 200 kilos of counterfeit meds seized in Capitol. The Phnom Penh Post, 2013 June 13. [cited 2013 Jul 29]. http://www.phnompenhpost.com/national/200-kilos-counterfeit-meds-seized-capital.

[9] Ruppe E, Hem S, Lath S, Gautier V, Ariey F, Sarthou JL (2009). CTX beta-lactamases in Escherichia Coli from Community-acquired urinary tracts Infections Cambodia. Emerg Infect Dis..

[10] Chheng K, Tarquinio S, Wuthiekanun V, Sin L, Thaipadungpanit J, Amornchai P (2009). Emergence of Community-associated methicillin resistant Staphylococcus aureus associated with pediatric infection in Cambodia. PLoS One..

[11] Vlieghe ER, Phe T, DeSmet B, Veng HC, Kham C, Lim K (2013). Bloodstream Infection among adults in Phnom Penh, Cambodia: key pathogens and resistance patterns. PLoS One.

[12] Bigdeli M, Annear PL (2009). Barriers to access and the purchasing function of health equity funds: lessons from Cambodia. Bull World Health Organ..

[13] Bauer AW, Kirby WM, Sherris JC, Turck M (1966). Antibiotic susceptibility testing by a standardized single disk method. Tech Bull Regist Med Technol..

[14] CLSI (2006). Performance standards for antimicrobial susceptibility testing: sixteenth informational supplement.

[15] CLSI (2008). Performance standards for antimicrobial susceptibility testing: eighteenth informational supplement.

[16] CLSI (2013). Performance standards for antimicrobial susceptibility testing: twenty-third informational supplement.

[17] Sasaki T, Hirai I, Niki M, Nakamura T, Komalamisra C, Maipanich W (2010). High prevalence of CTX-M b-lactamase-producing Enterobacteriaceae in stool specimens obtained from healthy individuals in Thailand. J Antimicrob Chemother.

[18] Ruppe E, Hem S, Lath S, Gautier V, Ariey F, Sarthou JL (2009). CTX-M β-Lactamases in Escherichia coli from Community-acquired Urinary Tract Infections, Cambodia. Emerg Infect Dis.

[19] Chareonkul C, Khun VL, Boonshuyar C (2002). Rational drug use in cambodia: study of three pilot health centers in Kampong Thom Province. Southeast Asian J Trop Med Public Health.

[20] Charani E, Cooke J, Holmes A (2010). Antibiotic stewardship programmes--what's missing?. J Antimicrob Chemother..

[21] Golding GR, Levett P, McDonald R, Irvine J, Nsungu M, Woods S (2010). A Comparison of risk factors associated with community-associated methicillin-resistant and –susceptible *Staphylococcus aureus* infections in remote communities. Epidemiol Infect..

[22] Tong SY, McDonald M, Holt D, Currie B (2008). Global implications of the emergence of community-associated methicillin-resistant *Staphylococcus aureus* in Indigenous populations. Clin Infect Dis..

[23] United States Center for Disease Control (US CDC) [internet]. CDC/NHSN Protocol Corrections, Clarification, and Additions. Updated 2013 Jan [cited 2012 Jul 29]. http://www.cdc.gov/nhsn/pdfs/pscmanual/17pscnosinfdef_current.pdf

[24] Liu C, Bayer A, Cosgrove S, Daum R, Fridkin S, Gorwitz R (2011). Clinical practice guidelines by the Infectious Disease Society of America for the treatment of Methicillin-resistant *Staphylococcus aureus* infections in adults and children: executive summary. Clin Infect Dis..

[25] Appelbaum PC (2007). Reduced glycopeptides susceptibility in methicillin-resistant Staphylococcus aureus (MRSA). Int J Antimicrob Agents..

[26] Petersen LR (2009). Bad bugs, no drugs: no ESCAPE revisited. Clin Infect Dis..

[27] Bush K (2013). Proliferation and significance of clinically relevant β-lactamases. Ann. N.Y. Acad. Sci..

[28] D’Costa VM, King C, Kalan L, Morar M, Sung W, Schwarz C (2011). Antibiotic resistance is ancient. Nature..

[29] Daikos GL, Markogiannakis A (2011). Carbapenemase-producing Klebsiella pneumonia: (when) might we still consider treating with carbapenems?. Clin Microbiol Infect..

[30] Bergen P, Landersdorfer C, Lee H, Li J, Nation R (2012). “Old” Antibiotics for emerging multidrug-resistant bacteria. Curr Opin Infect Dis.

[31] Polwhichai P, Dejsirilert S, Panpetch S, Sawanpanyalert P, Aswapokee N, Mootsikapun P (2009). Antimicrobial resistance of Escherichia coli isolated from urine in Thailand from 2000 to 2005. J Med Assoc Thai.

[32] Rawat D, Nair D (2010). Extended-spectrum Beta-lactamases in Gram Negative Bacteria. J Glob Infect Dis.

[33] Bush K, Jacoby G (2010). Updated Functional Classification of β-Lactamases. Antimicrob Agents Chemother.

[34] Kanj S, Kanafani Z (2011). Current Concepts in Antimicrobial Therapy Against Resistant Gram-Negative Organisms: Extended-Spectrum β-Lactamase-Producing Enterobacteriaceae, Carbapenem-Resistant Enterobacteriaceae, and Multidrug-Resistant *Pseudomonas aeruginosa*. Mayo Clin Proc.

